# Facilitated Visual Interpretation of Scores in Principal Component Analysis by Bioactivity-Labeling of ^1^H-NMR Spectra—Metabolomics Investigation and Identification of a New α-Glucosidase Inhibitor in Radix Astragali

**DOI:** 10.3390/molecules22030411

**Published:** 2017-03-06

**Authors:** Yueqiu Liu, Nils T. Nyberg, Anna K. Jäger, Dan Staerk

**Affiliations:** Department of Drug Design and Pharmacology, Faculty of Health and Medical Sciences, University of Copenhagen, Universitetsparken 2, DK-2100 Copenhagen, Denmark; yueqiuliu@gmail.com (Y.L.); nils.nyberg@bruker.com (N.T.N.); anna.jager@sund.ku.dk (A.K.J.)

**Keywords:** Radix Astragali, ^1^H-NMR spectroscopy, metabolomics, multivariate data analysis, type 2 diabetes, α-glucosidase inhibition assay

## Abstract

Radix Astragali is a component of several traditional medicines used for the treatment of type 2 diabetes in China. Radix Astragali is known to contain isoflavones, which inhibit α-glucosidase in the small intestines, and thus lowers the blood glucose levels. In this study, 21 samples obtained from different regions of China were extracted with ethyl acetate, then the IC_50_-values were determined, and the crude extracts were analyzed by ^1^H-NMR spectroscopy. A principal component analysis of the ^1^H-NMR spectra labeled with their IC_50_-values, that is, bioactivity-labeled ^1^H-NMR spectra, showed a clear correlation between spectral profiles and the α-glucosidase inhibitory activity. The loading plot and LC-HRMS/NMR of microfractions indicated that previously unknown long chain ferulates could be partly responsible for the observed antidiabetic activity of Radix Astragali. Subsequent preparative scale isolation revealed a compound not previously reported, linoleyl ferulate (**1**), showing α-glucosidase inhibitory activity (IC_50_ 0.5 mM) at a level comparable to the previously studied isoflavones. A closely related analogue, hexadecyl ferulate (**2**), did not show significant inhibitory activity, and the double bonds in the alcohol part of **1** seem to be important structural features for the α-glucosidase inhibitory activity. This proof of concept study demonstrates that bioactivity-labeling of the ^1^H-NMR spectral data of crude extracts allows global and nonselective identification of individual constituents contributing to the crude extract’s bioactivity.

## 1. Introduction

α-Glucosidase, an enzyme found in the brush border of the small intestines, hydrolyses terminal non-reducing (1→4)-linked α-d-glucose residues of di- to oligosaccharides. Inhibitors of α-glucosidase decrease the rate at which dietary carbohydrates are hydrolyzed, leading to lowered blood glucose levels [1,2]. α-Glucosidase inhibitors, like acarbose and miglitol, are used for treatment of patients with type 2 diabetes, and studies suggest that they can also be used to prevent impaired glucose tolerance to develop into type 2 diabetes [2,3,4]. Recently, compiled data show that more than 300 million people have diabetes worldwide, and this number is expected to increase to more than 500 million in 2030 [5]. China has the world’s largest diabetes epidemic with 11.6% of all Chinese adults, or 114 million people, having diabetes [5,6], and it is estimated that about 50% of the population is prediabetic [6]. It has been reported that α-glucosidase inhibitors play an important role in the antidiabetic activity of many traditional herbal preparations used for treatment of diabetes in China [7]. Investigations of bioactive constituents in complex extracts are laborious and time consuming, but new bioassay-coupled hyphenated NMR techniques for accelerated characterization of these bioactive metabolites have recently been developed [8,9,10].

Radix Astragali, *Astragalus membranaceus* (Fisch.) Bge. var. *mongholicus* (Bge.) Hsiao or *Astragalus membranaceus* (Fisch.) Bge [11,12], is referred to as “Huangqi” in China, and belongs to the Leguminosae family. It is a traditional Chinese medicine included in both the Chinese and European Pharmacopoeias, and is widely used for several therapeutic purposes, including the treatment of diabetes. More than 20 formulations of Radix Astragali are commercially available and widely used in China. More than half of these, such as “Tang mai kang ke li” and “Jing shi jiang tang pian”, contain Radix Astragali as the main ingredient. Most scientific papers reporting α-glucosidase inhibitory activity of Radix Astragali focus on flavonoids, and the inhibitory activity has mainly been ascribed to isoflavones, such as biochanin A, calycosin, and formononetin [13,14,15]. However, a literature search for compounds that inhibit α-glucosidase reveals that essentially all classes of natural products can be potential candidates, and thus there still might be other compounds with α-glucosidase inhibitory activity to discover. The aim of the current study was to perform a global and non-selective (in terms of chemical classes) investigation of α-glucosidase inhibitors in Radix Astragali. NMR-based metabolomics is in this context a useful technique as it is relatively unbiased towards different compound classes, allows for easy and robust sample preparation, and is quantitative (i.e., it has a linear response factor with linearity between concentration and signal strength) [16,17]. The covariance between inhibitory activities in the enzyme-based assay and the ^1^H-NMR spectroscopic profiles can easily be identified by color-labeling the samples exhibiting the highest inhibitory effect. This allows, in combination with hyphenated MS and NMR techniques [18,19], an unambiguous structural characterization of the constituents corresponding to the observed loadings.

## 2. Results and Discussion

^1^H-NMR fingerprints of Radix Astragali samples from different regions of China were color-labeled according to their α-glucosidase inhibitory activity. This allowed facilitated visual interpretation of scores from the principal component analysis (PCA), and thereby allowed identification of PCA loadings corresponding to ^1^H-NMR signals from α-glucosidase inhibitors.

### 2.1. Sample Preparation, IC_50_ Assessment, and ^1^H-NMR Data Acquisition

A total of 21 samples of Radix Astragali (Table 1) were obtained and extracted with three solvents of different polarity, that is, methanol, ethyl acetate, and dichloromethane, with the aim of extracting different classes of natural products. Neither the methanol nor the dichloromethane extracts showed pronounced α-glucosidase inhibition (data not shown), whereas the ethyl acetate extracts showed inhibition for most of the samples (Table 1).

The IC_50_-values ranged from 14 μg/mL to 1.4 mg/mL (Table 1), but for four of the samples, 50% inhibition was not obtained within the tested concentration range (inhibition curves in Supplementary Materials, Figure S1). The previously identified isoflavones with α-glucosidase activity, that is, biochanin A, calycosin, and formononetin (Supplementary Materials, Figure S2), and glycosides of these [13,14], were expected to be extracted with ethyl acetate and the large variation in activity is in line with a targeted LC-MS study [20] that showed considerable variation in the amounts of isoflavones among a set of 44 commercial samples of Radix Astragali.

**Figure 1:**
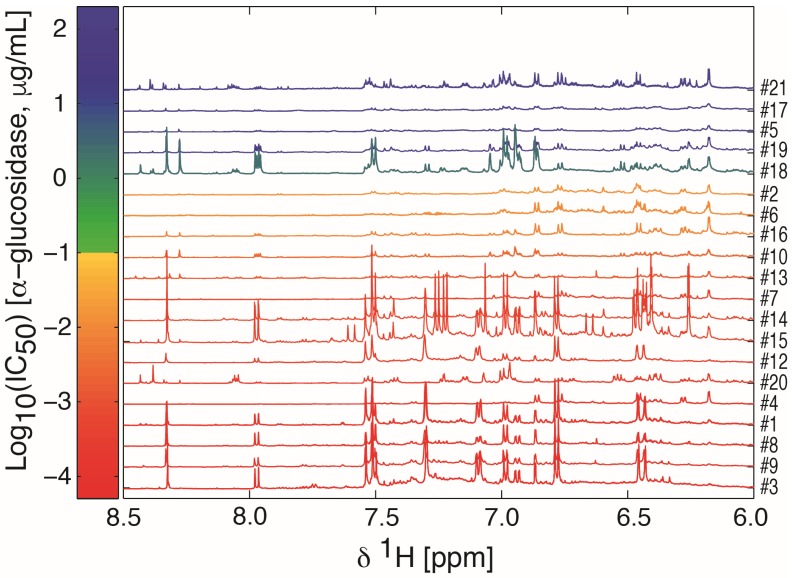
An expansion of 600 MHz ^1^H-NMR spectra of ethyl acetate extracts of Radix Astragali samples dissolved in DMSO-*d*_6_. The color and order of the spectra reflects the activity in the α-glucosidase inhibition assay according to the bar to the left. The number to the right is the number of the samples according to Table 1. Active extracts are those with IC_50_ values < 100 μg/mL and extracts with low activity are those with IC_50_ values ≥ 100 μg/mL. The non-active extract #19 showed only 30% inhibition at 5 mg/mL, and the remaining non-active extracts were arbitrarily set to 10 mg/mL for plotting purposes. The full spectral range 0–9 ppm can be seen in Supplementary Materials, Figure S3.

The dried ethyl acetate extracts were dissolved in deuterated dimethyl sulfoxide (DMSO-*d*_6_) and analyzed by 600 MHz ^1^H-NMR spectroscopy. The spectra were color-coded according to their IC_50_-value in the α-glucosidase inhibition assay, that is, bioactivity-labeled, and sorted according to this (Figure 1 and Supplementary Materials, Figure S3). The ^1^H-NMR spectra were dominated by signals in the aliphatic region, indicating large amounts of compounds with unsaturated fatty acids or long chain alcohols, including signals from terminal methyl groups below 1 ppm, the methylene envelope around 1.3 ppm, and signals from strongly coupled protons on *sp*^2^-carbons around 5.3 ppm (Supplementary Materials, Figure S3). Residual water and solvent signals were observed at 3.3 and 2.5 ppm, respectively. An expansion of the aromatic region (Figure 1) showed signals attributable to the flavonoids shown in Supplementary Materials, Figure S2 as well as signals attributable to caffeic or ferulic acid moieties.

### 2.2. Multivariate Data Analysis

**Figure 2:**
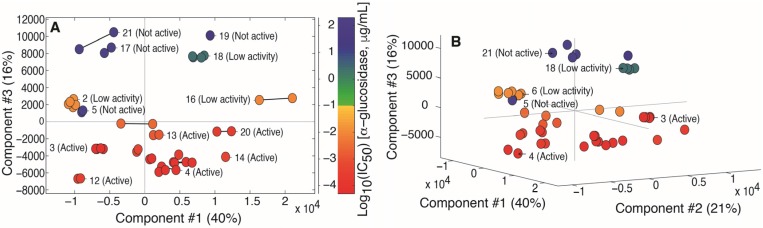
(**A**) Principal component analysis (PCA) scores plot of component **#3** vs. **#1**. Each marker represents one spectrum and solid lines between markers connect spectra of the same extract. The color of the markers represents the activity of the extracts in the α-glucosidase inhibition assay as described in the legend of Figure 1 and the color bar to the right. The numbers in parentheses are the percentage of the original variance that was captured in the specific component; (**B**) PCA scores plot of component **#3** vs. **#2** vs. **#1**. Each marker represents one spectrum. The color represents the activity of the extracts in the α-glucosidase assay as described in the legend of Figure 1 (red = active samples, blue = non-active samples).

A principal component analysis (PCA) [21] of the ^1^H-NMR spectra showed that samples with the highest α-glucosidase inhibitory activity also had the lowest scores in the third principal component (Figure 2A), and thus had a different spectral profile compared to samples with higher IC_50_-values. The number of relevant components was determined to be 3 by cross-validation, and the last component explained 16% of the original variation after Pareto scaling of the data. Neither origin, way of cultivation or age of the samples could be used to rationalize the scores and clustering of the spectra, even when considering all three components (data not shown). The distances between replicate and duplicate spectra were in all cases small compared to the overall differences between samples. This indicated that the sample preparation, the time between preparation and measurements of NMR spectra, and the data acquisition procedure did not affect the results significantly.

**Figure 3:**
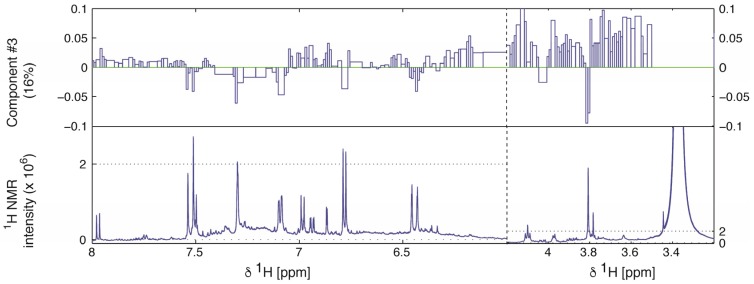
Two selected ranges of PCA-loading values of component **#3** (**top row**) compared to ^1^H-NMR spectra of the most active extract (sample 3). The vertical scaling of the loadings are the same in both ranges, whereas the vertical scaling in the ^1^H-NMR spectrum is indicated by the horizontal grid line.

The loadings suggested that the main differences between the samples in components **#1** and **#2** are varying amounts and different types of extracted fatty acids or long-chain alcohols (Supplementary Materials, Figure S4). A detailed comparison of loadings for principal component **#3** with spectra from the most active extract indicated that a series of intense signals in the aromatic region and a signal at 3.8 ppm might be correlated with the α-glucosidase inhibitory activity (Figure 3).

**Figure 4:**
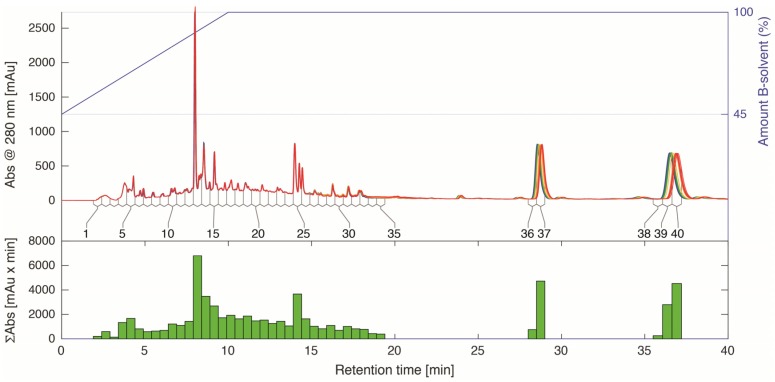
HPLC-chromatograms from a time-sliced fractionation of 20 repetitive analytical-scale separations (each corresponding to 0.5 mg dry weight on-column) of Radix Astragali sample #3 extract (**top**); Chromatographic profiles at 280 nm are overlaid with the first separation in blue color and the last in red color (intermediate separations in green and yellow). The fraction numbers of the 40 fractions are indicated under the chromatographic profiles and the gradient elution program on the right ordinate. The sums of UV-intensities are shown as bars plotted along the retention time axis (**bottom**).

These ^1^H-NMR signals did not quite match the spin system of the previously identified α-glucosidase inhibitors biochanin A, calycosin, and formononetin (Supplementary Materials, Figure S2). Thus, to further explore which chemical components could explain the observed loadings, RP-HPLC eluate of sample 3, was subjected to time-sliced fractionation. A gradient elution was used for the separation (Figure 4) and the elution continued at 100% B-solvent (95% acetonitrile with 0.1% formic acid) until everything was considered to be eluted. The fraction collector was set up to continuously capture fractions in the first part of the chromatographic profile and over two late-eluting components (Figure 4).

**Figure 5:**
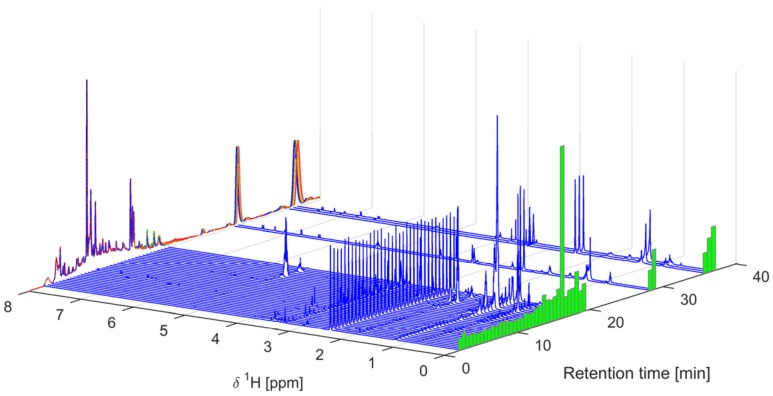
^1^H-NMR spectra of the 40 captured fractions dissolved in DMSO-*d*_6_ and plotted on the retention time axis according to their collection. The corresponding UV-chromatograms (first separation in blue color, last in red, intermediate in green and yellow, 20 in total) are plotted along the left-hand side and the summed NMR intensities are plotted as green bars on the right-hand side. The NMR-data were normalized so that the residual DMSO-signal (at 2.50 ppm) is of equal area for all spectra.

**Figure 6:**
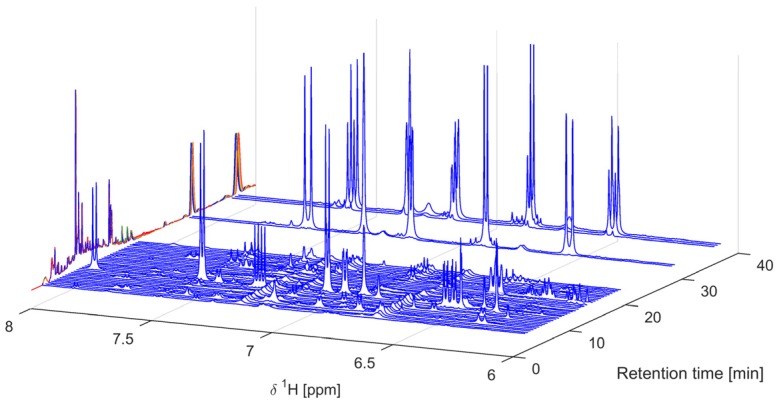
Expansion of ^1^H-NMR spectra of the 40 fractions dissolved in DMSO-*d*_6_ and plotted on the retention time axis according to their collection. The corresponding UV-chromatograms (first in blue color, last in red, intermediate in green and yellow, 20 in total) are plotted along the left-hand side.

A total of 40 fractions were collected, dried in vacuo, reconstituted in DMSO-*d*_6,_ and analyzed by NMR (Figure 5 and Figure 6).

### 2.3. Identification of α-Glucosidase Inhibitors

From the above data, it was obvious that the pattern in the aromatic region of principal component **#3** could be explained by either of the two late eluting peaks (Figure 4 and Figure 5, fraction 36–37 and fraction 38–40) that both showed identical signals in this range. The ^1^H-NMR spectra (Figure 5 and Figure 6) indicated that the late-eluting compounds were ferulic acids esterified with different long-chain alcohols. This was confirmed by further MS and NMR analyses (*vide infra*). A LC-MS analysis with electrospray ionization inlet operated in positive ion mode showed the major HPLC peak in the first part of the chromatogram (Figure 4, fraction 13) to have masses corresponding to the expected isoflavones formononetin (*m*/*z* 269.1), calycosin (*m*/*z* 285.1), and biochanin A (*m*/*z* 285.1). By adding up the total NMR signal intensity (green bars to the right in Figure 5), it could also be concluded that the two substituted ferulic acids (Figure 4 and Figure 5, fraction 36–37 and fraction 38–40) and the fatty acid or long-chain alcohol eluted around 16 min (Figure 5, fraction 29), constituted most of the extracted and fractionated material. The NMR-signal was in this respect more informative than the UV-trace (Figure 4) due to the linear response factor obtained with ^1^H-NMR. The total UV-absorbance of the corresponding fractions underestimated the amount of material due to lack of suitable chromophores representing the whole molecule.

### 2.4. LC-HRMS and NMR Analysis of HPLC Fractions

**Figure 7:**
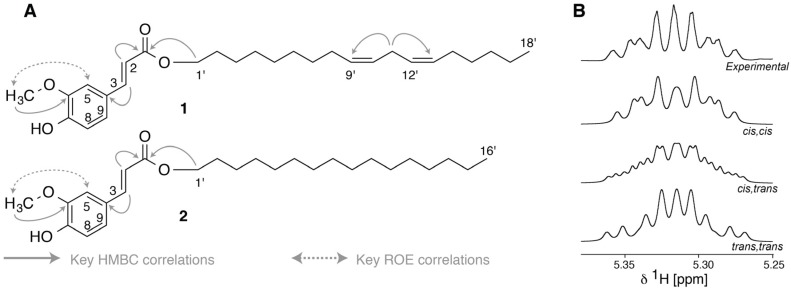
(**A**) Structure of linoleyl ferulate (**1**) and hexadecyl ferrulate (**2**) and selected HMBC and ROESY correlations; (**B**) Experimental (top) and simulated spectra (600 MHz) of the olefinic methine protons of the spin system -CH_2_-C***H***=C***H***-CH_2_-C***H***=C***H***-CH_2_ where the coupling is 7 Hz to the external methylenes (^3^*J*_H-8,H-9_ and ^3^*J*_H-13,H-14_) and 6.6 Hz to the central methylene (^3^*J*_H-10,H-11_ and ^3^*J*_H-11,H-12_), whereas the olefinic methine protons are coupled with 10 Hz (*cis*, *cis*), 10 and 16 Hz (*cis*, *trans*) and 16 Hz (*trans*, *trans*). The chemical shift values of the methine protons were set to 5.333, 5.297, 5.295 and 5.335 ppm.

Compound **1** (Figure 7A) showed an [M + H]^+^ ion with *m*/*z* 443.3152, which suggested the molecular formula C_28_H_42_O_4_. The ^1^H-NMR spectrum showed three aromatic protons of a 1,2,4-trisubstituted benzene [(δ 6.78, d, 8.1 Hz, H-8), (δ 7.09, dd, 8.1 and 1.7 Hz, H-9) and (δ 7.28, d, 1.7 Hz, H-5)] and two *trans*-coupled olefinic protons of an α,β-unsaturated carbonyl group [(δ 6.43, d 15.9 Hz, H-2) and (δ 7.52, d, 15.9 Hz, H-3)]. Key HMBC correlations from 6-OCH_3_ (δ 3.8, s) to C-6 (δ 148.2), H-2 to C-1 (δ 166.7) and H-3 to C-4 (δ 124.3) as well as a ROESY correlation from 6-OCH_3_ to H-5, established the ferulic acid moiety of **1** (Figure 7A). The two non-conjugated double bonds of the long chain alcohol moiety were both *cis* based on spin simulations, *vide infra*, and were identified by the apparent triplet with typical 6.6 Hz vicinal coupling for the intervening H-11′ (δ 2.71, t, ^3^*J*_H-10,H-11_ = ^3^*J*_H-11,H-12_ = 6.6 Hz), and HMBC correlations from H-11′ to C-9′ (δ 129.6) and C-13′ (δ 129.6). The actual position of the two double bonds could not be established by NMR spectroscopy, and the alcohol moiety was therefore cleaved by a base-catalyzed hydrolysis of the ester. It was subsequently analyzed by GC-MS, where a peak (r.t. 7.23 min) with molecular ion *m*/*z* 266 was identified as linoleyl alcohol by comparison with an authentic sample. Compound **1** in fraction 37 was therefore identified as linoleyl ferulate, which has not been reported previously. ^1^H- and ^13^C-NMR data are given in Table 2 and key ROESY and HMBC correlations are shown in Figure 7A. The all *cis* configuration of the double bonds was confirmed by spin system simulations using NMR-SIM, where *cis* coupling constants of 10 Hz for the double bonds fitted the experimental NMR data best (Figure 7B). Compound **2** (Figure 7A) showed an [M + H]^+^ ion with *m*/*z* 419.3145, which suggested the molecular formula C_26_H_42_O_4_. The structure was solved by NMR to be hexadecyl ferulate (**2**). Tabulated ^1^H and ^13^C chemical shifts of **2** are presented in Table 2, and key HMBC and ROESY correlations are shown in Figure 7A.

Alkyl ferulates have previously been found in different roots, and they seem to be important for suberization and wound healing of tubers [22,23,24]. They have not been reported from Radix Astragali before and no previous report on linoleyl ferulate (**1**) was found. Interestingly, similar ferulate esters, but with triple bonds in a shorter side chain, have been found in a thistle that has traditionally been used for treatment of diabetes [25].

### 2.5. α-Glucosidase Inhibitory Activity of Active Compounds

To assess the α-glucosidase inhibitory activity of **1** and **2**, they were collected in larger amounts by preparative-scale HPLC and the IC_50_-values were determined in the α-glucosidase inhibition assay. Linoleyl ferulate (**1**) showed inhibitory activity with an IC_50_ of 0.51 ± 0.04 mM. Interestingly, hexadecyl ferulate (**2**) did not show inhibitory activity (Supplementary Material, Figure S5) even though they share the same core structure. The IC_50_-value of (**1**) was in the same range as those previously reported for the isoflavones formononetin (0.5 mM) and biochanin A (0.3 mM) [13].

## 3. Materials and Methods

### 3.1. Plant Material and Extraction

A total of 21 samples of Radix Astragali were collected from different places in Gansu, Sichuan, Shanxi and Neimenggu provinces of China (Table 1). Voucher specimens (HQ101-HQ121) were deposited at Northwest Genuine Medicinal Materials Planting Cooperative (Jingyuan, Gansu, China). Powdered material from each plant was macerated in ethyl acetate (Sigma-Aldrich, St. Louis, MO, USA) and sonicated for 30 min. Subsequently, the mixture was shaken for one hour and left overnight. The next day, the mixture was shaken for another 10 min and filtered. The filtrate was evaporated to dryness under reduced pressure at 35 °C using a Savant SPD121P speed vacuum concentrator (Thermo Scientific, Waltham, MA, USA).

### 3.2. ^1^H-NMR Analysis of Mixtures

Dry samples were dissolved in DMSO-*d*_6_ (Sigma-Aldrich, St. Louis, MO, USA) to a final concentration of 50 mg/mL. The samples were sonicated for 20 min and then centrifuged at 13,000 rpm for 5 min. The supernatant (30 μL) of each sample was transferred into 1.7-mm o.d. NMR tubes. NMR experiments were performed on a Bruker Avance III 600 MHz NMR spectrometer (^1^H operating frequency 600.13 MHz) equipped with a Bruker SampleJet sample changer and a cryogenically cooled gradient inverse triple-resonance 1.7-mm TCI probe-head (Bruker Biospin, Rheinstetten, Germany). The ^1^H-NMR spectra were acquired at 25 °C using 30°-pulses, spectral widths of 12 kHz (20 ppm), and acquisition times of 5.4 s with additional relaxation delays of 1.0 s. Samples 1, 2, 3, 5, 6, 7, 9, 13, 16, 17, 18, 20 and 21 were prepared and analyzed in duplicate, and four of these (samples 1, 3, 6 and 18) were analyzed twice with at least one day between analyses. Three extracts were prepared once and analyzed once (samples 14, 15 and 19). Spectra of extract 11 were excluded due to instrumental problems during acquisition, leading to a total of 45 spectra acquired for multivariate analysis.

### 3.3. Data Analysis and Principal Component Analysis

The FIDs (128 k data points) of the ^1^H-NMR spectra were Fourier transformed to 64 k data points after exponential multiplication with a line broadening factor of 0.3 Hz (Topspin, version 3.2, Bruker BioSpin, Rheinstetten, Germany). The zeroth order phase correction parameters were automatically determined, baselines corrected (polynomials of degree 5 fitted to signal free ranges in the spectra) and spectra calibrated to the residual DMSO-signal at 2.50 ppm. The processed spectra were imported and further handled in Matlab (version R2013a, The MathWorks, Inc., Natick, MA, USA) using in-house written routines. The data were integrated in the range 0–9 ppm, excluding residual DMSO and water signals (2.45–2.56 and 3.20–3.52 ppm, respectively). The width and number of integration limits were initially determined by dividing the spectral range into units with a width of 0.01 ppm. Integrated ranges that were highly correlated (*R*^2^ > 0.95) and adjacent to each other were subsequently merged (iteratively) resulting in 437 ranges with a mean width of 0.02 ppm. The principal component analysis was performed with PLS_Toolbox (version 7.9.5, Eigenvector Research, Inc., Manson, WA, USA) after Pareto scaling of the data (mean centering and dividing by the square root of the variation of the respective variable). The number of relevant components was determined to be 3 by cross-validation. For this, the spectra were divided into 10 groups where spectra of the same samples were kept in the same groups. The first three PCA-components explained 77% of the variance (40%, 21%, and 16% for PC1, PC2 and PC3, respectively).

### 3.4. α-Glucosidase Inhibition Assay

The α-glucosidase inhibitory activity was determined in 96-well microplates according to the previously reported method [26]. Briefly, the extract was dissolved in 100 μL 0.1 M sodium phosphate buffer (pH 7.5, 0.02% NaN_3_) containing 10% DMSO. The IC_50_ values of the extracts were assessed using a dilution series starting at a concentration of 5 mg/mL, adding 80 μL α-glucosidase solution (type I, from *Saccharomyces cerevisiae*) in phosphate buffer (2.0 U/mL), and incubating at 28 °C for 10 min before 20 μL substrate solution of *p*-nitrophenyl α-d-glucopyranoside (10 mM in phosphate buffer) was added. Enzyme inhibition was determined by measuring the absorbance of the *p*-nitrophenol cleavage product at 405 nm for 35 min with a Multiskan FC microplate photometer controlled by SkanIt ver. 2.5.1 software (Thermo Scientific, Waltham, MA, USA).

### 3.5. Time-Sliced HPLC Separation

Chromatographic separation of the most active extract (sample 3) of Radix Astragali was performed with an Agilent 1200 series instrument (Santa Clara, CA, USA), consisting of a G1311A quaternary pump, a G1316A thermostatted column compartment, a G1315C diode array detector, a G1367C high-performance auto sampler, and a G1364C fraction collector. The separation was performed at 40 °C using a Phenomenex C_18_(2) Luna column (150 × 4.6 mm i.d., 3 μm, 100 Å; Phenomenex Inc., Torrance, CA, USA) with a flow rate of 0.5 mL/min. The aqueous eluent (A) consisted of water/acetonitrile (95:5, *v*/*v*) and the organic eluent (B) consisted of acetonitrile/water (95:5, *v*/*v*), both added 0.1% formic acid. The extract was separated 20 times, where each injection corresponded to 0.5 mg crude extract and time slice fractionated into 40 vials using the gradient: 0 min, 45% B; 10 min, 100% B; 45 min, 100% B. The fractions were dried, then dissolved in DMSO-*d*_6_ and analyzed by ^1^H-NMR with the same parameters as the analyses of the crude extracts.

### 3.6. LC-HRMS and NMR Analysis

HRMS data were obtained by a LC-HRMS system that by all practical means was identical to the Agilent-system described above, a Bruker micrOTOF-Q II mass spectrometer equipped with an electrospray ionization source (Bruker Daltonik GmbH, Bremen, Germany) operated in the positive ionization mode at 200 °C with a corona potential of 4 kV, a nebulizer pressure of 2.0 bar, and a drying gas flow of 7 L/min. Two-dimensional COSY, ROESY, HSQC, H2BC and HMBC NMR spectra (Bruker’s standard pulse sequences, 600.13 for ^1^H, 150.90 for ^13^C) were recorded on the above-mentioned instrument with ^1^H spectral widths of 12 ppm, and either 170 (HSQC, H2BC) or 240 ppm (HMBC) for ^13^C. The number of data points was 2 k in F2 and 128 (HMBC, H2BC, and ROESY), 256 (HSQC), or 512 (COSY) in F1. ROESY was obtained with 300 ms spinlock, HMBC with 62.5 ms evolution delay for *^n^J*_CH_ of 8 Hz, H2BC with 22 ms for evolution of *J*_HH_ and HSQC was optimized for a ^1^*J*_CH_ of 145 Hz. Relaxation delays were set to 1.0 s, except for the ROESY experiment, where the relaxation delay was set to 2.0 s. Data processing was performed using Topspin, version 3.2 (Bruker BioSpin, Rheinstetten, Germany), and spin simulations were performed in NMR-SIM (Bruker BioSpin, Rheinstetten, Germany).

### 3.7. GC-MS Analysis of Alcohol Moieties

The amount of 0.1 mg isolated ester was dissolved in 0.2 mL of 2 M LiOH in methanol in a 2-mL vial. After the solution had been stirred for 2 h the product was extracted with heptane (3 × 300 μL). The organic upper layer was analyzed by GC-MS using a 30 m × 0.25 mm i.d., 0.25 μm Zebron ZB-50 GC capillary column (Phenomenex Inc., Torrance, CA, USA). The column oven was held at 80 °C for 1 min and then increased 20 °C per min to 290 °C, and maintained at 290 °C for 3 min. Injector and detector temperatures were 230 °C and 150 °C respectively. The carrier gas was helium and the flow rate was 1.0 mL/min. The long-chain alcohol moieties of **1** was identified based on comparison with an authentic sample (Sigma-Aldrich) and its EI-MS spectra by comparison with entries in the 2002 version of NIST/EPA/NIH Mass Spectral Library (NIST 02).

### 3.8. Preparative-Scale Isolation and Determination of IC_50_ Values

Compounds **1** and **2** were obtained by a preparative-scale HPLC system consisting of Agilent 1100 series instrument with two preparative solvent delivery units, a multiple wavelength detector, an auto sampler, and a fraction collector. The separation was performed at 40 °C using a Phenomenex Luna C_18_ column (250 mm × 21.2 mm i.d., 5 μm, 100 Å; Phenomenex Inc., Torrance, CA, USA) by gradient elution as described for the time-sliced fractionation. The isolated compounds were reconstituted in DMSO for determination of IC_50_ values in the α-glucosidase inhibition assay.

## 4. Conclusions

In conclusion, the ethyl acetate extracts of Radix Astragali from different geographical regions of China showed variations in α-glucosidase inhibitory activity. The multivariate data analysis of bioactivity-labeled ^1^H-NMR spectra revealed that spectral profiles correlated with the α-glucosidase inhibitory activity, which allowed identification of linoleyl ferulate (**1**) a previously unidentified α-glucosidase inhibitor in Radix Astragali. Furthermore, the difference in inhibitory activity between **1** (containing an unsaturated linoleyl side chain) and **2** (containing a saturated hexadecyl side chain) supports the results of a recent study, that showed oleic acid and linoleic acid had much higher α-glucosidase inhibitory activity than the saturated palmitic acid [27]. This study shows that bioactivity-labeling, that is, color coding, of ^1^H-NMR spectra, facilitates visual interpretation of scores from the principal component analysis. This method can easily be adopted for the study of other medicinal plants.

## Figures and Tables

**Table 1:** Radix Astragali samples investigated and the IC_50_-values determined in the α-glucosidase inhibition assay of ethyl acetate extracts. The three most active extracts are typeset in bold.

No.	Origin ^a^	Voucher ^b^	IC_50_ (µg/mL) ^c^
1	Xihe, Gansu (cultivated)	HQ106	19 ± 0
2	Kangding, Sichuan	HQ101	157 ± 13
**3**	**Dingxi, Gansu (cultivated)**	**HQ107**	**14 ± 1**
4	Songpan, Sichuan (cultivated)	HQ102	19 ± 1
5	Longxi, Gansu (cultivated, 3 years)	HQ111	>10,000 ^d^
6	Litang, Sichuan (wild, >3 years)	HQ103	136 ± 7
7	Litang, Sichuan (wild, 1–2 years)	HQ104	35 ± 1
**8**	**Weiyuan, Gansu (cultivated, 4–5 years)**	**HQ108**	**18 ± 4**
**9**	**Weiyuan, Gansu (cultivated, 2–3 years)**	**HQ109**	**17 ± 1**
10	Zhangxian, Gansu (cultivated)	HQ119	71 ± 2
11	Xiaojin, Sichuan (wild)	HQ105	107 ± 12
12	Dangchang, Gansu (cultivated)	HQ110	27 ± 7
13	Longxi, Gansu (cultivated)	HQ112	51 ± 2
14	Lixian, Gansu (cultivated, 3 years)	HQ113	34 ± 1
15	Minxian, Gansu (cultivated, 3 years)	HQ114	29 ± 1
16	Jingyuan, Gansu (cultivated, 2 years)	HQ115	129 ± 38
17	Jingyuan, Gansu (cultivated, 3–4 years)	HQ116	>10,000 ^d^
18	Zhangxian, Gansu (cultivated, 3 years)	HQ117	1457 ± 170
19	Zhangxian, Gansu (cultivated, 2 years)	HQ118	>5000 ^e^
20	Wutaishan, Shanxi (cultivated)	HQ120	26 ± 1
21	Neimenggu (cultivated)	HQ121	>10,000 ^d^

^a^ Province and county, cultivated or harvested in the wild, age of plant (if known); ^b^ Northwest Genuine Medicinal Materials Planting Cooperative (Jingyuan, Gansu, China); ^c^ IC_50_-values with 95% confidence intervals of the fitted parameter; ^d^ No inhibition at highest concentration (10 mg/mL); ^e^ 30% inhibition at 5 mg/mL.

**Table 2:** NMR spectroscopic data (DMSO-*d*_6_, 600 and 150 MHz for ^1^H and ^13^C, respectively) of compounds **1** and **2** acquired at 300 K.

Pos.	1		2	
	δ_C_^a^	δ_H_ (*J* in Hz) ^a,b^	δ_C_^a^	δ_H_ (*J* in Hz) ^a,b^
1	166.7		166.9	
2	114.4	6.43 d (15.9)	114.3	6.44 d (16.0)
3	144.8	7.52 d (15.9)	144.8	7.52 d (16.0)
4	124.3		123.1	
5	111.1	7.28 d (1.7)	110.8	7.29 d (1.7)
6	148.2		148.3	
7	149.2		149.1	
8	115.4	6.78 d (8.1)	115.3	6.78 d (8.1)
9	123.0	7.09 dd (8.1, 1.7)	123.1	7.09 dd (8.1, 1.7)
1′	63.7	4.09 t (6.7)	63.6	4.10 t (6.6)
2′	28.1	1.61 m	27.7	1.61 tt (7.3, 6.3)
3′	25.3	1.32 m	25.2	1.33 m
4′	28.6	1.26 overlap	28.6	1.22 overlap
5′	28.6	1.26 overlap	28.6	1.22 overlap
6′	28.6	1.26 overlap	28.6	1.22 overlap
7′	28.8	1.26 overlap	28.6	1.22 overlap
8′	26.5	1.99 overlap	28.6	1.22 overlap
9′	129.6	5.31 m	28.6	1.22 overlap
10′	127.7	5.28 m	28.6	1.22 overlap
11′	25.1	2.71 t (6.6)	28.6	1.22 overlap
12′	127.7	5.28 m	28.6	1.22 overlap
13′	129.6	5.31 m	28.6	1.22 overlap
14′	26.5	1.99 overlap	30.9	1.22 overlap
15′	28.8	1.26 overlap	22.0	1.24 overlap
16′	28.8	1.26 overlap	13.8	0.84 t (6.8)
17′	21.9	1.24 overlap	-	-
18′	13.9	0.85 t (6.8)	-	-
6-OCH_3_	55.7	3.80 s	55.5	3.80 s

^a^ Spectra referenced to residual DMSO signal at 2.50/39.51 ppm; ^b^ s = singlet, d = doublet, dd = double doublet, t = triplet, m = unresolved multiplet, overlap (for signals in the methylene envelope).

## Supplementary Materials

Supplementary materials are available online. Figure S1: α-glucosidase inhibition curves of crude extracts, Figure S2: Structures of expected isoflavones, Figure S3: ^1^H-NMR spectra of all samples, Figure S4: PCA loadings, and Figure S5: α-glucosidase inhibition curves of **1** and **2**.
