# Supplementary material for: Facilitated Visual Interpretation of Scores in Principal Component Analysis by Bioactivity-Labeling of 1H-NMR Spectra—Metabolomics Investigation and Identification of a New α-Glucosidase Inhibitor in Radix Astragali
Source: Molecules. 2017 Mar 6;22(3):411. doi: 10.3390/molecules22030411 (PMC6155417; doi:10.3390/molecules22030411)
Supplement: Supplementary file 1 [file molecules-22-00411-s001.pdf]

Supplementary Material for

Facilitated visual interpretation of scores in principal component analysis by bioactivity-labeling of ^{1}H NMR spectra - metabolomics investigation and identification of a new α-glucosidase inhibitor in Radix Astragali

Yueqiu Liu[a], Nils T. Nyberg[a], Anna K. Jäger[a], Dan Staerk[a,*]

[a]*Department of Drug Design and Pharmacology, Faculty of Health and Medical Sciences, University of Copenhagen, Universitetsparken 2, DK-2100 Copenhagen, Denmark*

[*]Corresponding author. Tel.: +45 35336177; fax: +45 35336001.

E-mail address: ds@sund.ku.dk (D. Staerk).

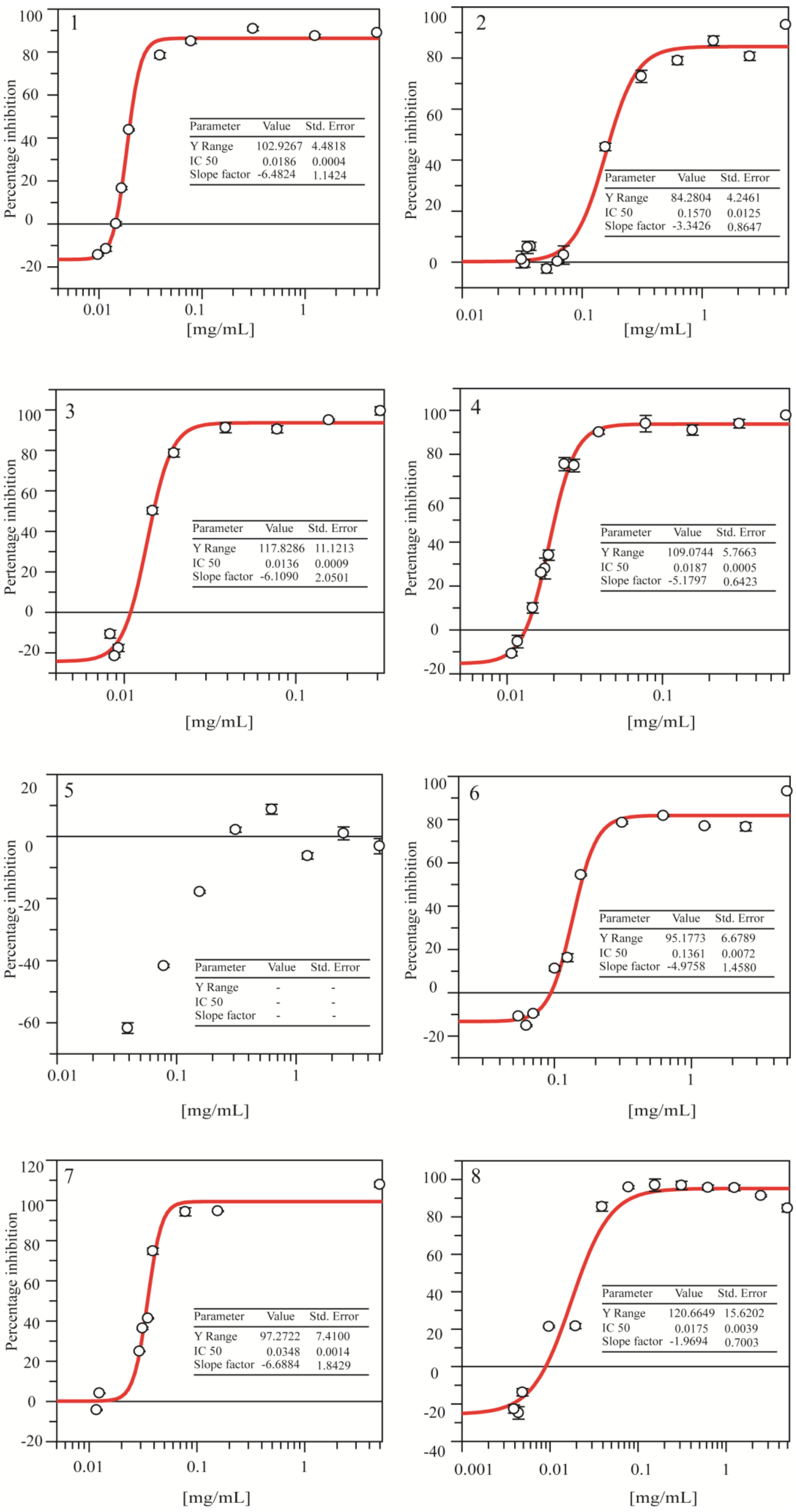

Figure S1. Inhibition curves of the ethyl acetate extracts of 21 samples of Radix Astragali in the α-glucosidase inhibition assay.

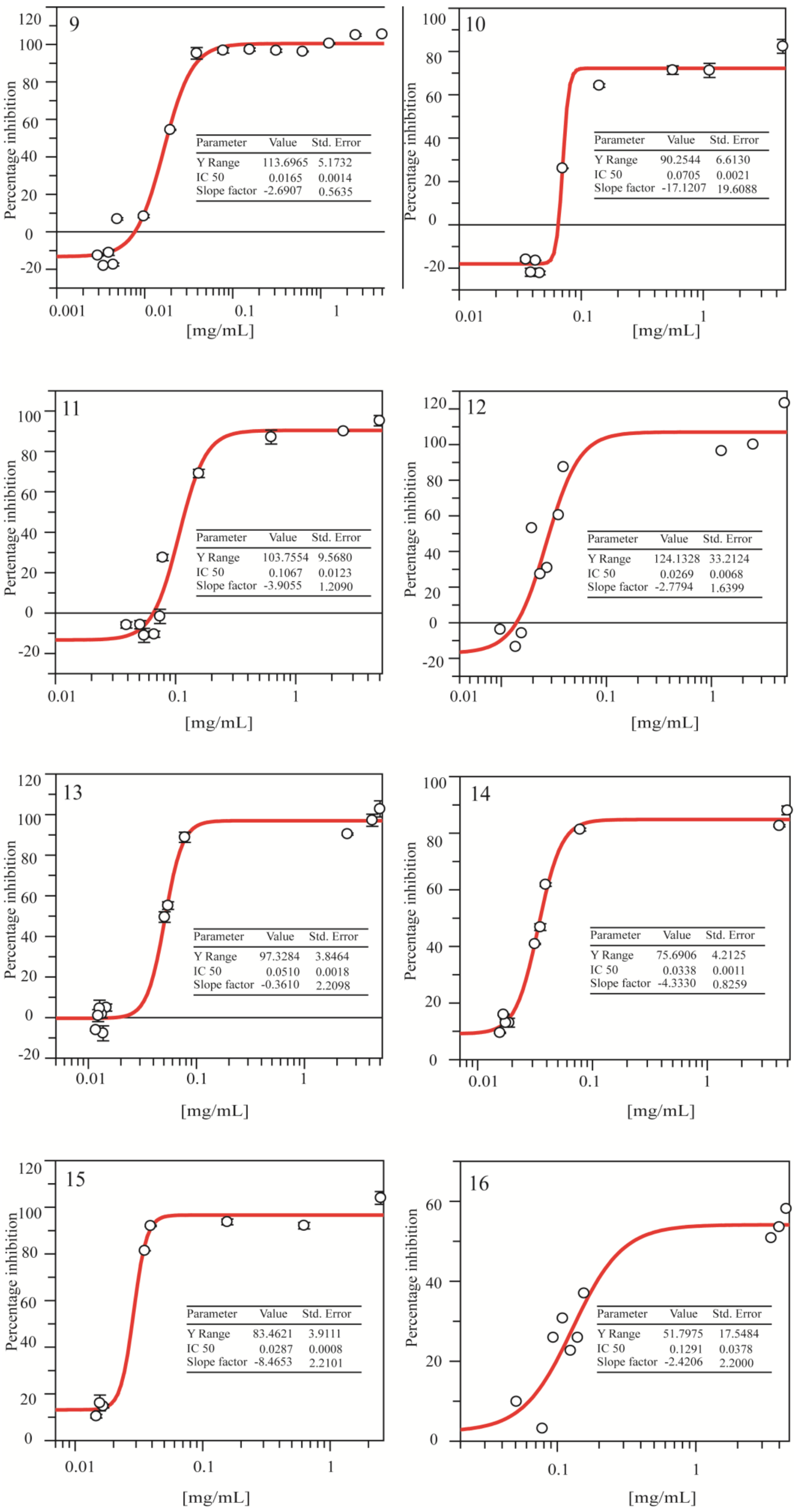

Figure S1 continued. Inhibition curves of the ethyl acetate extracts of 21 samples of Radix Astragali in the α-glucosidase inhibition assay.

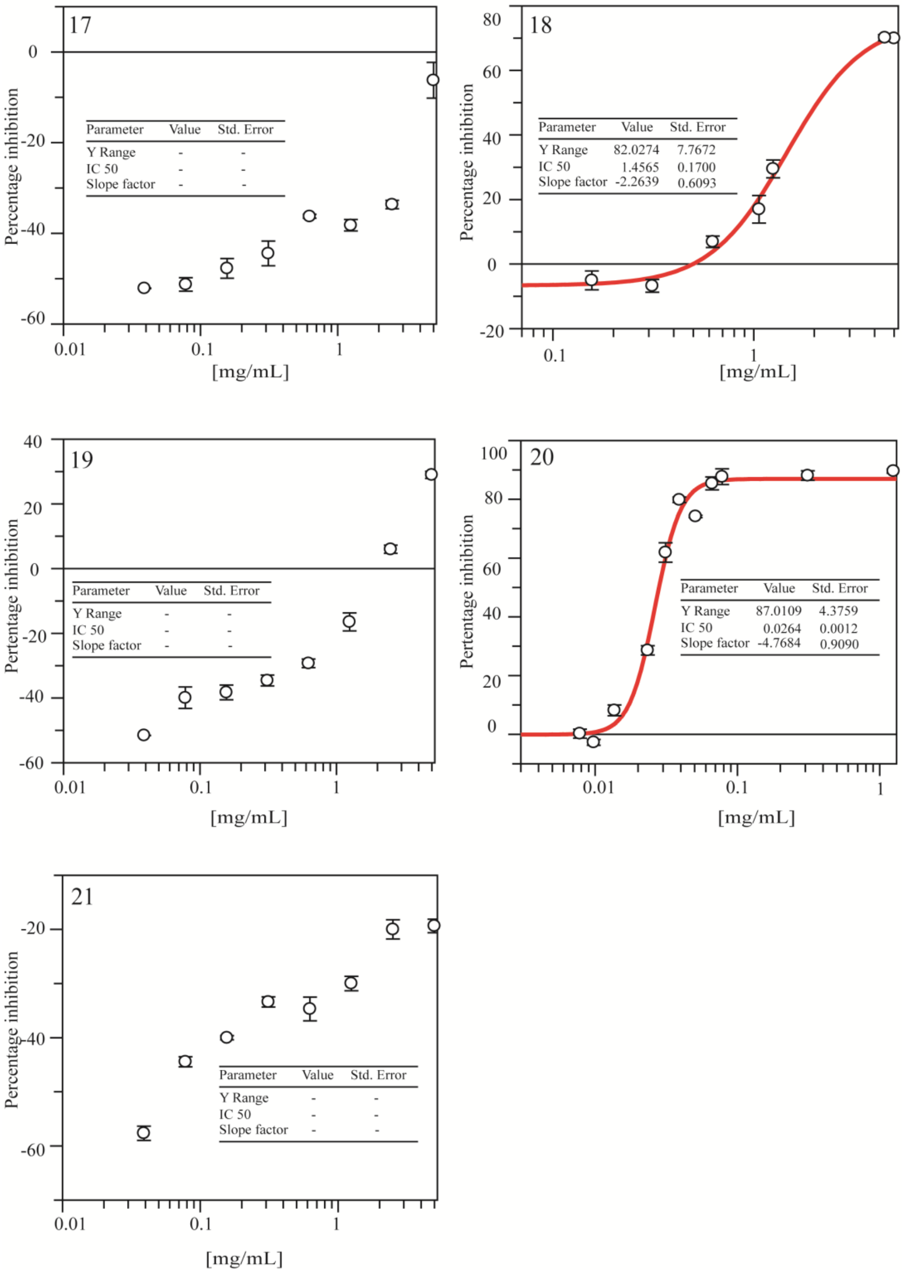

Figure S1 continued. Inhibition curves of the ethyl acetate extracts of 21 samples of Radix Astragali in the α-glucosidase inhibition assay.

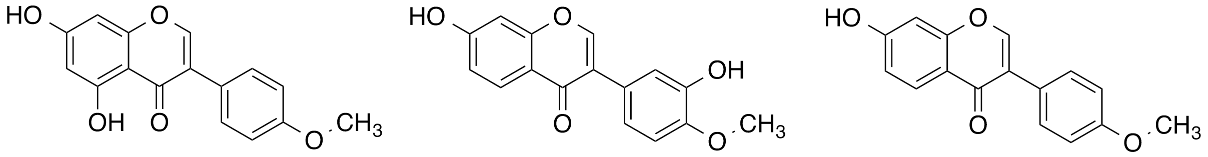

Biochanin A Calycosin Formononetin

Figure S2. Radix Astragali isoflavones with expected α-glucosidase inhibitory activity.

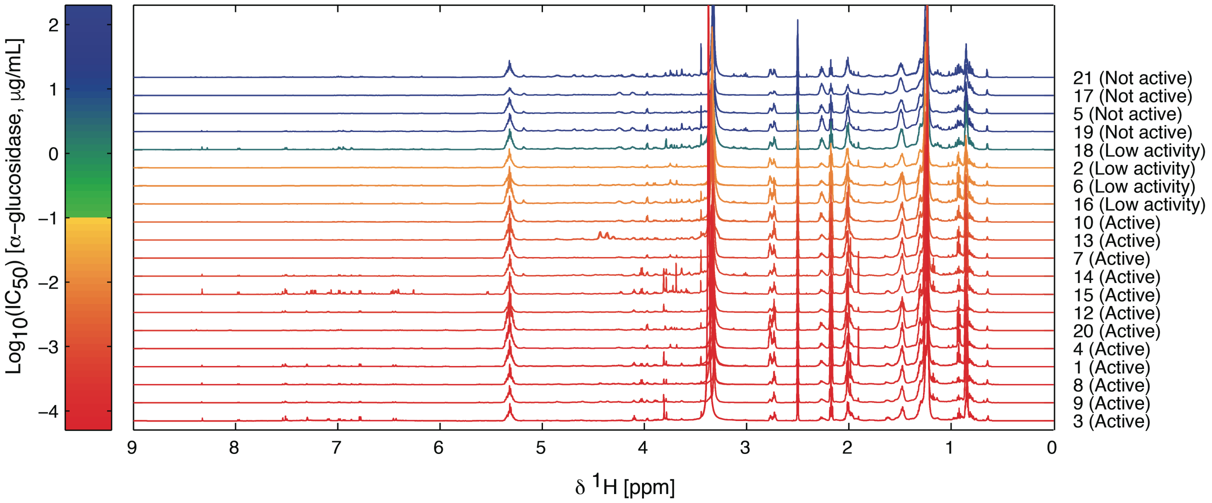

Figure S3. 600 MHz ^{1}H NMR spectra of ethyl acetate extracts of Radix Astragali samples dissolved in DMSO-d_6. The color and order of the spectra reflects the inhibitory activity in the α-glucosidase assay according to the bar to the left. The number to the right gives the number of the sample. Active extracts are those with IC_{50} values < 100 μg/mL and extracts with low activity are those with IC_{50} values ≥ 100 μg/mL. The non-active extract # 19 showed only 30 % inhibition at a concentration of 5 mg/mL and the remaining non-active extracts were arbitrarily set to 10 mg/mL for plotting purposes.

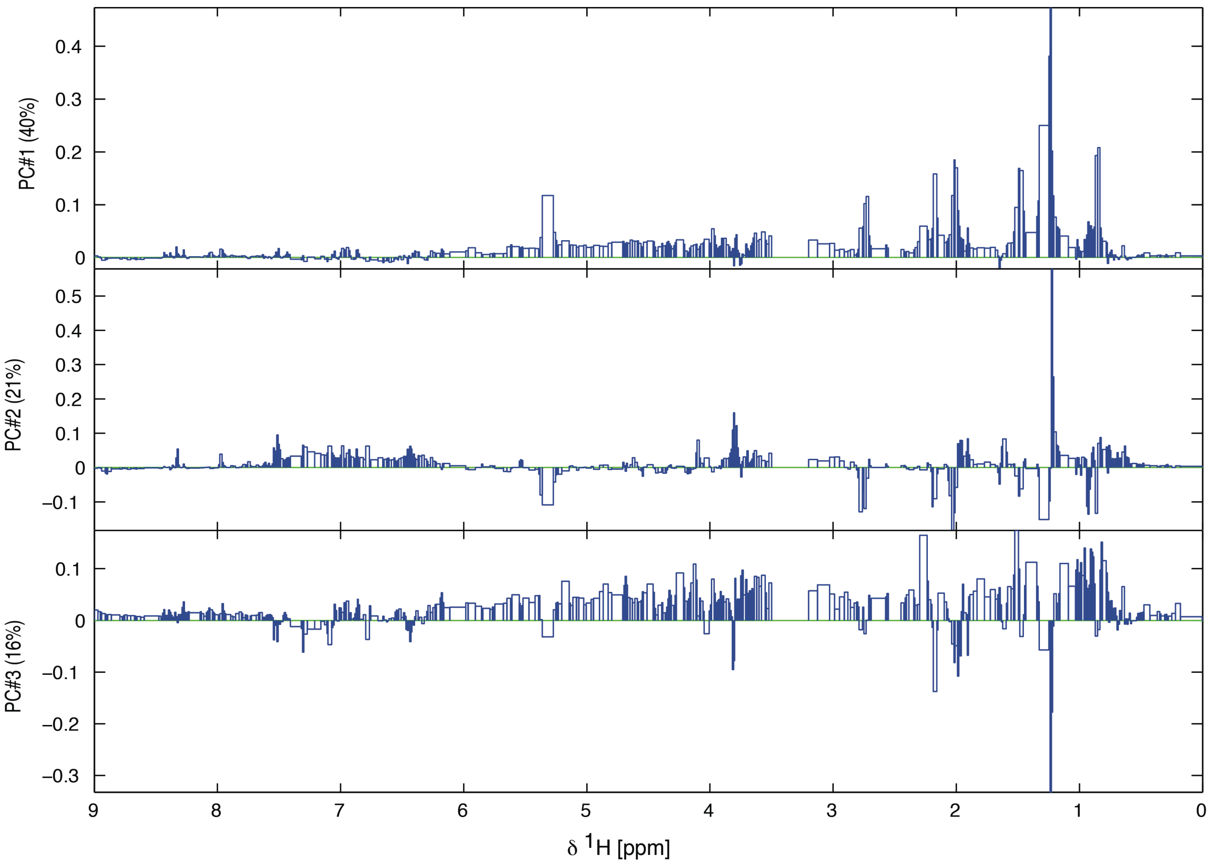

Figure S4. PCA-loading values for components #1, #2 and #3 plotted on the chemical shift axis.

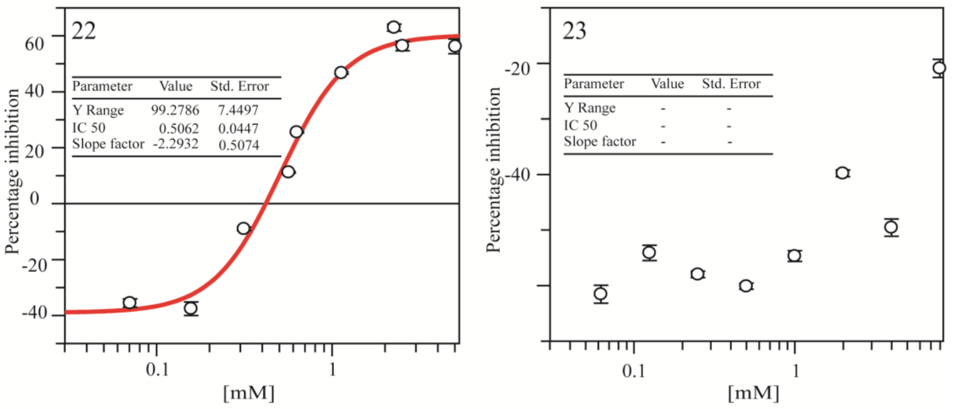

Figure S5. α-Glucosidase inhibition curves: linoleyl ferulate (**1**, sample 22) and hexadecyl ferulate (**2**, sample 23).
